# Phase II trial of delta-tocotrienol in neoadjuvant breast cancer with evaluation of treatment response using ctDNA

**DOI:** 10.1038/s41598-023-35362-7

**Published:** 2023-05-24

**Authors:** Ina Mathilde Kjær, Søren Kahns, Signe Timm, Rikke Fredslund Andersen, Jonna Skov Madsen, Erik Hugger Jakobsen, Tomasz Piotr Tabor, Anders Jakobsen, Troels Bechmann

**Affiliations:** 1 Department of Biochemistry and Immunology, Vejle Hospital, University Hospital of Southern Denmark, Vejle, Denmark; 2 Department of Oncology, Vejle Hospital, University Hospital of Southern Denmark, Vejle, Denmark; 3 Department of Regional Health Research, Faculty of Health Sciences, University of Southern Denmark, Odense, Denmark; 4 Department of Medicine, Hospital Soenderjylland, University Hospital of Southern Denmark, Aabenraa, Denmark; 5 Department of Pathology, Vejle Hospital, University Hospital of Southern Denmark, Vejle, Denmark; 6 Department of Pathology, Viborg Hospital, Regional Hospital Central Jutland, Viborg, Denmark; 7 Department of Oncology, Regional Hospital West Jutland, Herning, Denmark

**Keywords:** Breast cancer, Cancer therapy, Tumour biomarkers

## Abstract

Neoadjuvant treatment of breast cancer is applied to an increasing extent, but treatment response varies and side effects pose a challenge. The vitamin E isoform delta-tocotrienol might enhance the efficacy of chemotherapy and reduce the risk of side effects. The aim of this study was to investigate the clinical effect of delta-tocotrienol combined with standard neoadjuvant treatment and the possible association between detectable circulating tumor DNA (ctDNA) during and after neoadjuvant treatment with pathological treatment response. This open-label, randomized phase II trial included 80 women with newly diagnosed, histologically verified breast cancer randomized to standard neoadjuvant treatment alone or in combination with delta-tocotrienol. There was no difference in the response rate or frequency of serious adverse events between the two arms. We developed a multiplex digital droplet polymerase chain reaction (ddPCR) assay for the detection of ctDNA in breast cancer patients that targets a combination of two methylations specific for breast tissue (*LMX1B* and *ZNF296*) and one cancer specific methylation (*HOXA9*). The sensitivity of the assay increased when the cancer specific marker was combined with the ones specific to breast tissue (*p* < 0.001). The results did not show any association between ctDNA status and pathological treatment response, neither at midterm nor before surgery.

## Introduction

Neoadjuvant treatment of breast cancer is applied to an increasing extent with the aim of downstaging the tumor to allow for breast conserving surgery, especially in HER2 positive and triple negative breast cancer^1^. Treatment response, however, varies and side effects of the chemotherapy pose a challenge. The ability of vitamin E to enhance the efficacy of chemotherapy and reduce the risk of side effects has been a field of major interest. A recent systematic review concluded that while administration of the E-vitamin isoform alpha-tocopherol as complementary cancer treatment seems to reduce oral side effects, it may have a negative influence on survival rates due to reduced effectiveness of chemotherapy or radiotherapy. Hence, the use of alpha-tocopherol as complementary cancer treatment is not recommended^2^.

In contrast to tocopherols, the potential of the tocotrienol isoforms has been investigated in clinical studies to a much lesser extent^3^, but recent research indicates that tocotrienols, in particular the delta and gamma isoforms, may hold stronger anti-cancer potency^4^. In vitro and in vivo studies indicate that tocotrienol exerts an antineoplastic effect through complex pathways by inhibiting invasion, metastasizing and angiogenesis and by inducing apoptosis and autophagy, with no indications of harmful effects on normal cells^4^.

Only few studies have investigated the potential of tocotrienol in a clinical cancer setting^3,5–7^, and to our knowledge, only one clinical study investigating the effect of tocotrienol in combination with breast cancer treatment has been published, so far^7^. This was a non-randomized, placebo-controlled pilot trial with 240 early-stage breast cancer patients who received tamoxifen alone or in combination with tocotrienol (200 mg daily) for five years. The results did not show any association between tocotrienol administration and breast cancer specific survival, but the study had several limitations, including lack of randomization^7^. Thus, randomized clinical trials investigating the effect of tocotrienol in breast cancer treatment are warranted.

Response to neoadjuvant breast cancer treatment is known to vary, and there is a need for sensitive and valid methods for response evaluation to ensure adequate neoadjuvant treatment for the individual patient without inducing unnecessary side effects of ineffective treatment. Circulating tumor DNA (ctDNA) has been studied as prognostic and predictive biomarkers in early breast cancer patients undergoing neoadjuvant therapy. A recent study reports that persistence of ctDNA during neoadjuvant treatment is associated with poor tumor response^8^. A systematic review and meta-analysis published in 2022 investigated the prognostic value of ctDNA detection in patients with early breast cancer undergoing neoadjuvant treatment. The study concluded that presence of ctDNA at baseline and lack of ctDNA clearance after neoadjuvant treatment might be associated with decreased relapse free and overall survival^9^.

The primary aim of the present study was to investigate the clinical effect of delta-tocotrienol combined with standard neoadjuvant treatment in a randomized, controlled phase II trial in primary breast cancer. The secondary aim was to develop a universal droplet digital polymerase chain reaction (ddPCR) assay for detection of ctDNA and its possible association with treatment response and survival.

## Methods

### Patient eligibility and study design

This open label, randomized, parallel group, phase II trial was conducted at the Department of Oncology, Vejle Hospital, Denmark, and enrolled women with newly diagnosed, histologically verified breast cancer eligible for neoadjuvant treatment according to prevailing guidelines. The selection criteria are presented in Table 1. The patients were randomized 1:1 to either standard neoadjuvant treatment (Arm A) or standard neoadjuvant treatment in combination with delta-tocotrienol (Arm B). The hospital pharmacy generated the random allocation sequence. Randomization was computerized and stratified according to HER2 and ER-status based on a tumor biopsy (three strata). Patients were assigned to the interventions by the hospital’s clinical trial unit. All patients gave written and orally informed consent to participation. The study was conducted in accordance with the Helsinki Declaration. The protocol was approved by the Regional Committees on Health Research Ethics for Southern Denmark (S-20160009) and the Danish Medicines Agency (2,016,034,241). The study was registered with ClinicalTrials.gov (NCT02909751, 21/09/2016). It adheres to the Consolidated Standards of Reporting Trials (CONSORT)^10^ and good clinical practice (GCP).

**Table 1:** Eligibility criteria for the NeoToc study.

Inclusion criteria	Exclusion criteria
Histologically verified breast cancer (adenocarcinoma)	Bilateral breast cancer or suspected dissemination of breast cancer (evaluated by bilateral mammography, bone-scintigraphy, computer tomography of thorax and abdomen and positron emissions tomography)
Age ≥ 18 years	Mental or social conditions that could detain treatment or follow-up
Neoadjuvant treatment indicated according to departmental guidelines	Other simultaneous experimental treatment
Performance status 0–2 (Eastern Cooperative Oncology Group) and suited for surgery	Other malignant disease within five years (with the exception of non-melanoma skin cancer and carcinoma in situ cervicis uteri)
Normal heart function (left ventricle ejection fraction ≥ 50% evaluated by multiple gated acquisition/echocardiogram in patients receiving neoadjuvant trastuzumab)	Immunosuppressive treatment (with the exception of prednisolone administered in connection with neoadjuvant chemotherapy)
Normal function of bone marrow (hemoglobin ≥ 6 mmol/l, absolute neutrophil count ≥ 1.5 × 10^9^/l and thrombocytes ≥ 100 × 10^9^/l)	Vitamin or nutritional supplements (with the exception of multivitamin tablets or calcium and vitamin D tablets)
Normal liver function (bilirubin ≤ 1.5 × upper reference limit, alanine-aminotransferase ≤ 2.5 × upper reference limit and alcalic phosphatase ≤ 2.5 × upper reference limit)	Previous treatment with docetaxel, paclitaxel, epirubicin, cyclophosphamide, trastuzumab, pertuzumab or tocotrienol
Normal kidney function (creatinine ≤ upper reference limit or estimated glomerular filtration rate ≥ 50 ml/min)	Rheumatoid arthritis or other autoimmune disease
Presentation of a negative pregnancy test in fertile women and use of medically acceptable contraception during neoadjuvant treatment and three months after	Pregnancy or breastfeeding
Written and orally informed consent	Active or latent viral/bacterial infection
	Hypersensitivity to any of the active or auxiliary substances

### Treatment

Patients in both treatment arms received neoadjuvant chemotherapy according to prevailing guidelines, i.e. four cycles of epirubicin 90 mg/m^2^ intravenously (IV) combined with cyclophosphamide 600 mg/m^2^ IV every three weeks followed by four cycles of a taxane (docetaxel 100 mg/m^2^ IV every three weeks or paclitaxel 80 mg/m^2^ IV every week). Patients with biopsy verified HER2-positive breast cancer received four cycles of HER2 targeted treatment combined with a taxane followed by four cycles of epirubicin and cyclophosphamide. The HER2 targeted treatment was administered as trastuzumab alone (initially 8 mg/kg IV as saturation dose followed by 6 mg/kg IV every three weeks) or in combination with pertuzumab (initially 840 mg IV as saturation dose followed by 420 mg IV every three weeks). Patients in arm B received 300 mg oral delta-tocotrienol three times a day from the first day of chemotherapy until the day of surgery in addition to standard neoadjuvant treatment. The tocotrienol supplement was manufactured by American River Nutrition (USA) and contained 90% delta-tocotrienol. DuraPharma (Denmark) supplied the product (Traptol®). After neoadjuvant treatment, the patients underwent lumpectomy or mastectomy with sentinel node guided removal of axillary lymph nodes.

### Efficacy, toxicity and outcomes

Treatment response was based on histopathological assessment of the tissue removed at surgery. The graduation of pathological response was based on the Residual Cancer Burden (RCB) score (MD Anderson)^11^, classifying the response on a scale from RCB 0 (pathological complete response (pCR)) to RCB III (significant tumor burden). Adverse events were registered every three weeks the day before each cycle of chemotherapy according to the Common Terminology Criteria for Adverse Events 4.0 (CTCAE). Survival endpoints were overall survival (OS) and invasive disease free survival (IDFS) defined as the time from inclusion in the study until the case of an event. OS included death of any cause. IDFS included local or systemic breast cancer recurrence, diagnosis of other primary invasive cancer (with the exception of squamous or basal cell skin cancers) or death of any cause as defined in Proposal for standardized definitions for efficacy end points in adjuvant breast cancer trials: the STEEP system^12^.

### Blood samples, DNA extraction and ctDNA analysis

Blood samples were collected prior to the 5th cycle of neoadjuvant treatment (midterm) and before and after breast cancer surgery. The preoperative blood samples were obtained 0–18 days before surgery (after completion of neoadjuvant treatment), and the postoperative blood samples were obtained 4–46 days after surgery. Plasma isolation and DNA extraction was performed as described previously^5^. The genes encoding the LIM homeodomain transcription factor 1 beta (*LMX1B*) and Zinc finger protein 296 (*ZNF296*) have been reported to contain cytosine residues followed by a guanine nucleotide (CpGs) with a breast tissue specific methylation signature, and to be potent biomarkers for detection and monitoring of localized breast cancer^13^. Homeobox protein A9 (HOXA9) is a cancer specific marker with prognostic importance in several malignant tumors^14,15^.We designed a multiplex ddPCR assay targeting bisulfite converted DNA specific for 1) breast epithelium cells (*LMX1B* sense + *LMX1B* antisense + *ZNF296* sense assays) and 2) a general marker of cancer (*HOXA9* sense + *HOXA9* antisense).

The assays are described in detail in the supplementary material. Bisulfite converted DNA extracted from blood and breast tissue from healthy women was used to test the specificity of the multiplex assay (Figure S3). The limit of blank (LOB) was determined with a 95% confidence limit by analyzing plasma samples from healthy women (Figure S4). A blood sample was defined as ctDNA-positive when the signal for one or more of the targets described above was above the LOB. The ctDNA analysis of plasma samples was performed blinded to clinical outcomes.

### Statistical methods

The study was based on Simon's two-stage minimax design. The clinical assumption was that a pCR frequency ≥ 50% was interesting for further studies, whereas a frequency ≤ 30% was of no clinical relevance. With a significance level of 5% and a power of 80%, 19 patients should be enrolled in each arm in the first part of the study. If < 6 patients in arm B achieved pCR, the study would be terminated. Otherwise, enrollment would continue to a total of 39 patients in each arm. If > 16 of the patients in arm B achieved pCR, the treatment with delta-tocotrienol would be interesting for further studies. Thus, a total of 78 evaluable patients should be enrolled in the study. Balanced diagnostics for baseline covariates were estimated using standardized differences^16^. Standardized differences above 0.1 were considered imbalanced.

Data on delta-tocotrienol treatment were analysed as intention-to-treat according to pathological response and adverse events (grade 3–4). They were presented as contingency tables with frequencies and proportions and calculated as two-sided *p*-values corresponding to Fisher's exact test. RCB classification was categorized as RCB 0 (pCR) versus RCB I-III (no pCR), and analyses were stratified by ER/HER2-status. The sensitivity of the ctDNA assay targeting *HOXA9* alone and in combination with the markers *LMX1B* and *ZNF296* were presented as contingency tables with frequencies and proportions, and calculated as two-sided p-values using Fisher's exact test*.* Distributions of ctDNA positivity and pathological response were analysed at two time points, midterm and preoperatively, and also presented as contingency tables with frequencies and proportions, and two-sided p-values corresponding to Fisher's exact test. Due to low plasma volume in some preoperative blood samples (N = 6), worst and best case scenarios were estimated to explore the robustness of the findings. Post hoc analyses of RCB classification similar to Zhou et al.^8^ (RCB 0–I vs. RCB II-III) were performed. OS and IDFS were analysed according to delta-tocotrienol treatment and ctDNA positivity at three time points: midterm, preoperatively and postoperatively. Data were presented as overall survival rates and 10th percentile survival time for OS and IDFS, and visualized in Kaplan–Meier survival functions with corresponding p-values for log-rank test of equal survivor functions. All statistical analyses were performed in STATA 17 (STATA Corp., TX, USA), and p-values below 0.05 were considered statistically significant.

## Results

### Patient characteristics

**Figure 1:**
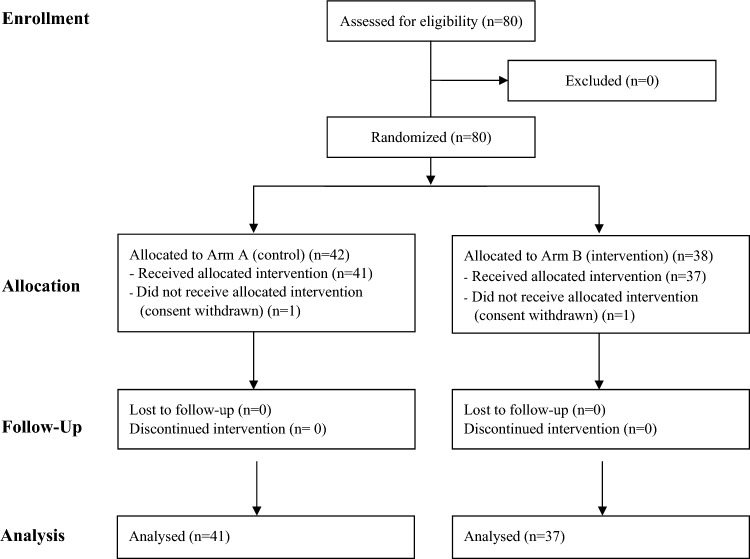
CONSORT flowchart of NeoToc: A randomized phase II trial of delta-tocotrienol in combination with standard treatment in neoadjuvant breast cancer treatment. The control group (Arm A) received standard neoadjuvant treatment. The intervention group (Arm B) received standard neoadjuvant treatment in combination with delta-tocotrienol.

A total of 80 patients with newly diagnosed breast cancer were enrolled from September 2016 to July 2018 at the Department of Oncology, Vejle Hospital. Interim analysis was conducted after enrollment of 19 patients in each arm. Results showed that 7 of 19 patients in Arm B had pCR and enrollment continued. The study flow is presented in Fig. 1. Forty-two patients were randomized to the control group (Arm A) and 38 to the intervention group (Arm B). The uneven number of participants in the two arms was caused by the stratification (ER and HER2 status). One patient in each group withdrew their consent before initiating neoadjuvant treatment. All other patients completed the study according to randomization group and no patients were lost to follow-up. Baseline patient characteristics are presented in Table 2. Standardized differences showed imbalances between the two arms in relation to ER-status, tumor size, tumor grade and pathological lymph nodes with the control arm having slightly more advanced disease.

**Table 2:** Baseline patient characteristics. Patients included in Arm A received standard neoadjuvant treatment according to existing guidelines. Patients included in Arm B received standard neoadjuvant treatment in combination with 300 mg oral delta-tocotrienol three times daily from the first day of chemotherapy until the day of surgery.

	Arm A (control group)	Arm B (intervention group)	All	Standardized differences**
Patients, N	41	37	78	
Age (mean, min–max)	54.2 (28.5–82.8)	54.4 (34.9–81.7)	54.7 (28.5–82.8)	0.03
Strata				
HER2 negative / ER positive	18 (44%)	13 (35%)	31 (40%)	− 0.15
HER2 negative / ER negative	9 (22%)	9 (24%)	18 (23%)	
HER2 positive	14 (34%)	15 (41%)	29 (37%)	
ER status (pre-treatment biopsy)^a^				
Positive (≥ 1%)	30 (73%)	21 (57%)	51 (65%)	0.37
Negative (0%)	11 (27%)	16 (43%)	27 (35%)	
HER2 status (pre-treatment biopsy)^b^				
Positive	14 (34%)	15 (41%)	29 (37%)	− 0.09
Negative	27 (66%)	22 (59%)	49 (63%)	
Tumor size (baseline MR)				
T1 ≤ 20 mm	8 (20%)	7 (19%)	15 (19%)	0.12
T2 21–50 mm	24 (59%)	25 (68%)	49 (63%)	
T3 > 50 mm	4 (10%)	1 (3%)	5 (6%)	
Missing	5 (12%)	4 (11%)	9 (12%)	
Pathological lymph nodes^c^				
Yes	36 (88%)	29 (78%)	65 (83%)	0.40
No	5 (12%)	8 (22%)	13 (17%)	
Tumor grade (pre-treatment biopsy)				
1	–	1 (3%)	1 (1%)	0.29
2	14 (34%)	15 (41%)	29 (37%)	
3	8 (20%)	10 (27%)	18 (23%)	
Unknown	19 (46%)	11 (30%)	30 (38%)	
Histological type (pre-treatment biopsy)				
Ductal	26 (63%)	23 (62%)	49 (63%)	− 0.03
Lobular	2 (5%)	1 (3%)	3 (4%)	
Other	13 (32%)	13 (35%)	26 (33%)	
Chemotherapy^d^*				
Yes	41 (100%)	37 (100%)	78 (100%)	N/A
No	–	–	–	
HER2 targeted treatment^e^*				
Yes	14 (34%)	15 (41%)	29 (37%)	− 0.09
No	27 (66%)	22 (59%)	49 (63%)	
Tocotrienol^f^*				
Yes	–	37 (100%)	37 (47%)	N/A
No	41 (100%)	–	41 (53%)	

^a^ER status: Estrogen receptor status of breast cancer tumor.

^b^HER2 status: Status of human epidermal growth factor receptor 2 (HER2) in breast cancer tumor evaluated by immunohistochemistry (IHC) and fluorescence in situ hybridization (FISH). Positive: IHC 3 + or IHC 2 + and FISH > 2. Negative: IHC 0 or IHC 1 + or IHC 2 + and FISH < 2.

^c^ Pathological lymph nodes defined as malignant cells in primary lymph node biopsy, malignant cells in sentinel lymph node preoperatively, or malignant cells in lymph node removed at time of breast cancer surgery.

^d^Chemotherapy: Four cycles of epirubicin 90 mg/m^2^ IV in combination with cyclophosphamide 600 mg/m^2^ IV every three weeks followed by four cycles of a taxane (docetaxel 100 mg/m^2^ IV every three weeks or paclitaxel 80 mg/m^2^ IV every week).

^e^HER2 targeted treatment: Patients with HER2 positivity of the breast cancer tissue (biopsy) received four cycles of HER2 targeted treatment in combination with taxane initially followed by four cycles of epirubicin and cyclophosphamide. The HER2 targeted treatment was administered as trastuzumab alone (initially 8 mg/kg IV as saturation dose followed by 6 mg/kg IV every three weeks) or in combination with pertuzumab (initially 840 mg IV as saturation dose followed by 420 mg IV every three weeks).

^f^Tocotrienol: In addition to standard treatment patients randomized to arm B received 300 mg oral delta-tocotrienol three times daily from the first day of chemotherapy until the day of surgery.

* Treatment registered as “yes” if one or more cycles were administered.

** Standardized differences above 0.1 were considered imbalanced.

### Response to treatment

Pathological response according to RCB classification is presented in Table 3. Two patients were diagnosed with lymph node metastasis in the sentinel node biopsy conducted prior to neoadjuvant treatment, which invalidates the accuracy of the RCB calculation^11^. Thus, 76 patients were included in the RCB analysis. Eighteen (18/39, 46%) and 15 patients (15/37, 41%) in the control and intervention group, respectively, achieved pCR (*p* = 0.65) (Table 3). Stratifying the analysis by ER and HER2 status also did not show any difference in pCR between the control and intervention arms (data not shown).

**Table 3:** Pathological complete response to neoadjuvant breast cancer treatment and serious adverse events during treatment. Patients in both arms received a standard neoadjuvant treatment regimen with delta-tocotrienol added in the intervention group.

	Yes	No	*P*-value
Pathological complete response (RCB 0)			
Arm B (Intervention) (n = 37)	15 (41%)	22 (59%)	0.65
Arm A (Control) (n = 39)	18 (46%)	21 (54%)	
Adverse events (grade 3 and 4)			
Arm B (Intervention) (n = 37)	13 (35%)	24 (65%)	0.81
Arm A (Control) (n = 41)	13 (32%)	28 (68%)	

Pathological response evaluated by histopathological assessment of the surgically removed tissue with graduation according to the Residual Cancer Burden (RCB) Calculator (MD Anderson^11^). Pathological complete response corresponds to RCB 0. Serious adverse events (grade 3 and 4) according to Common Terminology Criteria for Adverse Events 4.0 (CTCAE).

### Survival

**Figure 2:**
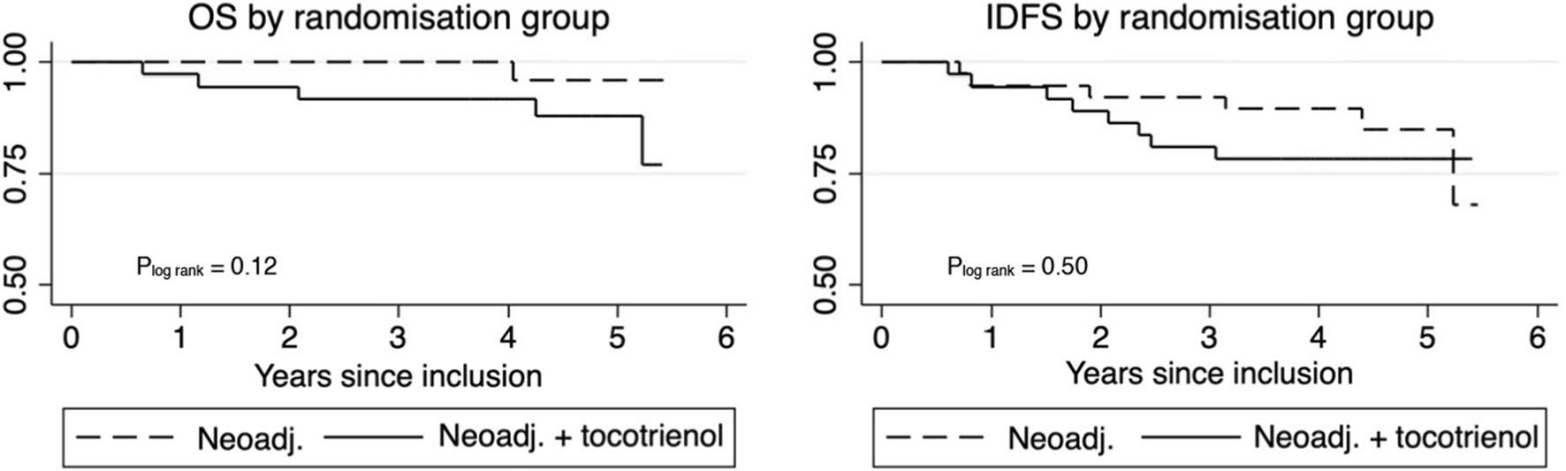
Overall survival (OS) and invasive disease free survival (IDFS) from the date of inclusion (September 2016–July 2018) in patients enrolled in the NeoToc study receiving standard neoadjuvant breast cancer treatment only and in combination with delta-tocotrienol.

The follow-up period ended April 1, 2022. The total amount of risk time was 335.4 person-years corresponding to a median follow-up time of 4.41 years (IQR 3.98–5.01 years). During the study period, 92% survived (N = 70) and 82% (N = 62) survived disease free. The 10^th^ percentile of survival time for OS in the control arm was 3.67 years (95% CI 3.64–3.75) compared to 3.93 years (95% CI 3.65–4.08) in the intervention arm. For IDFS, the 10^th^ percentile was 3.67 (95% CI 3.64–3.73) and 3.90 years (95% CI 3.65–4.14) in the control and intervention arm, respectively. Hence, there was no difference in OS and IDFS between the two arms (Fig. 2).

### Adverse events

Serious adverse events (grade 3 and 4 according to CTCAE 4.0) were observed in 13 patients in the control group (13/41, 32%) and in 13 patients in the intervention group (13/37, 35%) with no difference between the two arms (*p* = 0.81) (Table 3).

### ctDNA

#### Sensitivity and specificity of assay

We developed a multiplex ddPCR assay targeting ctDNA with a methylation signature specific for breast tissue (*LMX1B* and *ZNF296*) and one general marker of cancer (*HOXA9*).

In order to increase sensitivity, we included both sense and antisense targets of *LMX1B* and *HOXA9* in the multiplex assay. For the *ZNF296* assay only a sense target was included, as we did not succeed in designing a specific antisense ZNF296 assay. The assays displayed specificity towards DNA extracted from breast tissue only and not towards whole blood and plasma samples from healthy individuals (Figure S3 and S4).

The multiplex assay was used for the analysis of plasma samples available from 76 patients at midterm and from 63 patients preoperatively. A total of 19 midterm and 9 preoperative samples displayed positivity for the cancer specific marker *HOXA9* alone. The fraction of positive samples increased from 19/76 to 40/76 at midterm (*p* < 0.001) and from 9/63 to 21/63 (*p* = 0.013) preoperatively by simultaneously targeting the cancer and breast tissue specific ctDNA markers (Table 4).

**Table 4:** Sensitivity of circulating tumor DNA multiplex assay targeting the cancer specific marker *HOXA9* alone and in combination with the breast tissue specific markers *LMX1B* and *ZNF296.*

	*HOXA9* alone	*LMX1B* or *ZNF296* or* HOXA9*	*P*-value
Midterm (n = 76)			
+	19 (25%)	40 (53%)	< 0.001
−	57 (75%)	36 (47%)	
Preoperative (n = 63)			
+	9 (14%)	21 (33%)	0.013
−	54 (86%)	42 (67%)	

#### CtDNA as a biomarker for treatment response

**Figure 3:**
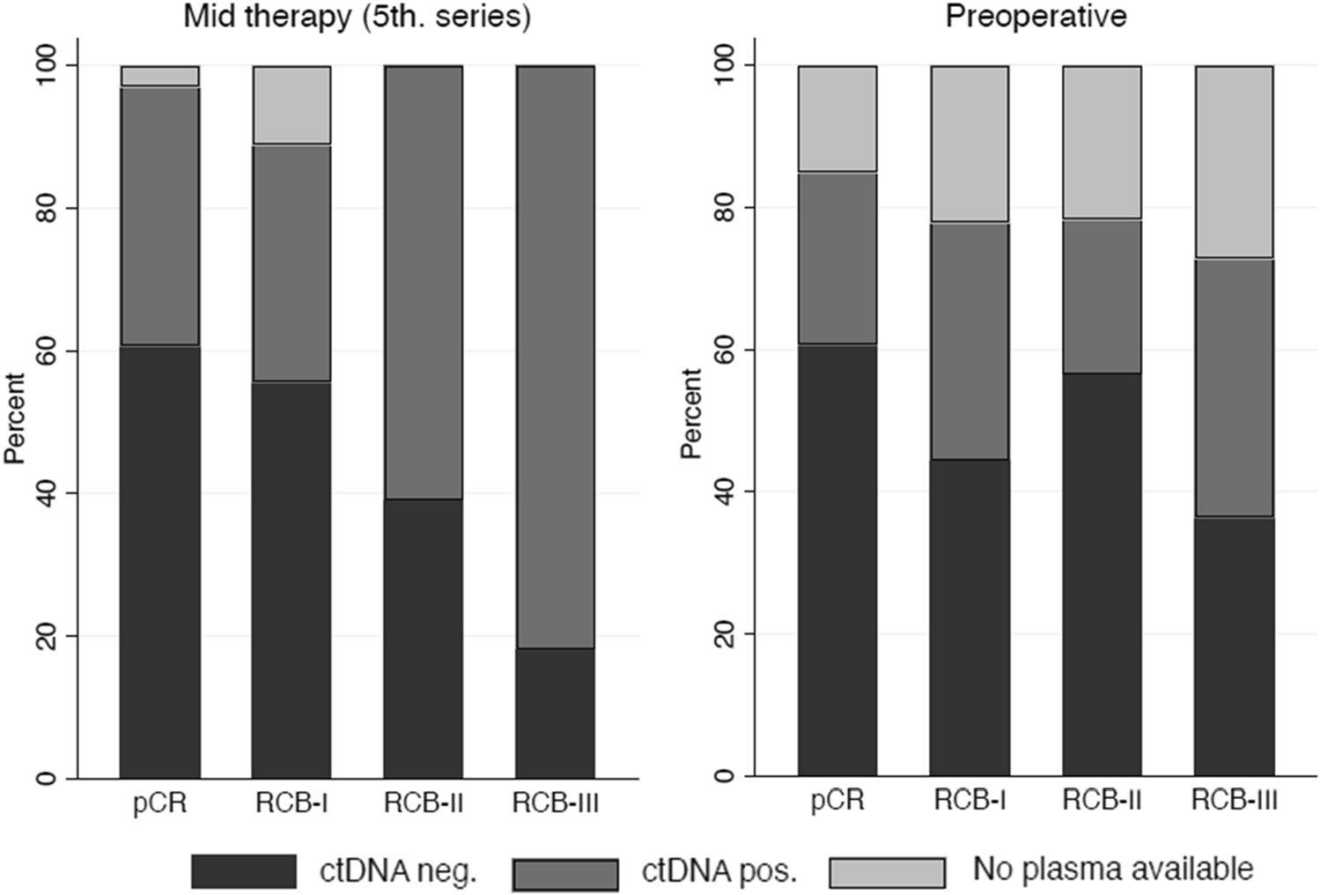
CtDNA status at midterm and preoperatively in relation to pathological response to neoadjuvant breast cancer treatment according to histopathological assessment of the surgically removed tissue with grading according to the Residual Cancer Burden (RCB) Calculator (MD Anderson^11^). Pathological complete response (pCR) corresponds to RCB 0.

Data on RCB class were available for 74 of the patients with midterm blood samples. Among patients with ctDNA positivity at midterm 12 achieved pCR (12/38, 32%), whereas 20 of 36 patients (56%) with ctDNA negativity at midterm achieved pCR (*p* = 0.06) (Table 5). Preoperative blood samples were available from 63 patients. Thirty-eight percent (8/21) of patients with ctDNA positivity before surgery achieved pCR, whereas 48% (20/42) of those with ctDNA negativity achieved pCR (*p* = 0.6) (Table 5). No difference was observed between the two treatment arms (data not shown). The preoperative blood sample from six patients had reduced plasma volume, which may have resulted in false negative ctDNA status. A worst case and best case scenario analysis did not change the outcome (data not shown). Figure 3 depicts the ctDNA status of the patients at midterm and preoperatively in relation to the RCB class obtained at breast cancer surgery. Survival analysis showed no association between ctDNA status and OS and IDFS, respectively, neither at midterm, preoperatively nor postoperatively (Supplementary material).

**Table 5:** Pathological complete response according to presence of circulating tumor DNA (ctDNA) evaluated at midterm (before the 5th cycle of neoadjuvant treatment) and preoperatively in breast cancer patients.

	Pathological complete response		
	Yes	No	*P*-value
Midterm			
ctDNA + (n = 38)	12 (32%)	26 (68%)	0.06
ctDNA − (n = 36)	20 (56%)	16 (44%)	
Preoperative			
ctDNA + (n = 21)	8 (38%)	13 (62%)	0.6
ctDNA − (n = 42)	20 (48%)	22 (52%)	

CtDNA status was evaluated using a multiplex droplet digital polymerase chain reaction assay targeting a combination of two breast tissue specific (*LMX1B* and *ZNF296*) and one cancer specific methylation (*HOXA9*). A blood sample was defined as ctDNA positive when the signal of one or more of the targets was above the limit of blank.

Post hoc analysis was conducted using RCB classification similar to Zhou et al.^8^ (RCB 0/I vs. RCB II/III). It showed that 68% (23/34) of the patients with poor response to neoadjuvant treatment (RCB II/III) had ctDNA positivity at midterm, whereas 38% (15/40) of those responding to neoadjuvant treatment (RCB 0/I) had ctDNA positivity at midterm (*p* = 0.012). After completion of neoadjuvant treatment, prior to surgery, 36% (10/28) of the patients with poor response (RCB II/III) had a ctDNA positive blood sample as compared to 31% (11/35) of the responding patients (RCB 0/I) (*p* = 0.79).

## Discussion

The primary aim of this randomized, controlled, phase II trial was to investigate the clinical effect of delta-tocotrienol in combination with standard neoadjuvant treatment in primary breast cancer. The results showed no difference in response rate to neoadjuvant breast cancer treatment between patients who received delta-tocotrienol and the control group. Also, there was no difference in the frequency of serious adverse events, OS, and IDFS between the two arms. Thus, in this study we found that delta-tocotrienol did not enhance the efficacy of neoadjuvant treatment and did not reduce the frequency of side effects.

Although the study was conducted as a randomized, controlled trial, it has several limitations. Despite the randomization, balanced diagnostics showed imbalance with regard to ER-status, tumor size, tumor grade and pathological lymph nodes, indicating slightly more advanced disease stage in the control group. However, since no difference in outcome was observed between the arms, and the imbalance points towards more advanced disease in the control group, we expect the implications of the imbalance to have very limited significance in relation to the overall outcome of this study. The lack of blinding might have induced bias, as it cannot be ruled out that patients randomized to the control arm self-administered a vitamin E product without reporting it. Also, the unblinded design might have caused information bias in the reporting of side effects. The risk of a type two error was 20% and as the Simon’s two stage design is exploratory as opposed to confirmatory, the results call for further investigation. Therefore, based on this study and previous clinical trials across cancer types^3,5–7^, the potential of tocotrienol to enhance the efficacy of cancer treatment and reduce side effects is undecided, and well-planned blinded, randomized clinical trials are warranted.

The secondary aim of this study was to develop an assay for detection of ctDNA in breast cancer. In general, ctDNA detection in patients with a low tumor burden is challenging due to low concentrations of ctDNA^17^. The assay sensitivity has been reported to increase with an increasing number of independent ctDNA targets^18^ and when bisulfite converted DNA is targeted in both the sense and the antisense direction^19^. We developed a multiplex ctDNA ddPCR assay including two breast tissue specific (*LMX1B* and *ZNF296*) and one cancer specific target (*HOXA9*). Two of them (*LMX1B* and *HOXA9*) were targeted in both the sense and the antisense direction. Compared to the sensitivity of the cancer specific marker (*HOXA9*) alone, the addition of two breast tissue specific markers increased the sensitivity of the assay significantly and more than doubled the number of ctDNA positive samples. This indicates that addition of tissue specific markers to multiplex assays can increase the performance of ctDNA assays significantly, which should be considered in future studies aiming at designing ctDNA assays for application in early stage cancer with a low tumor burden.

Based on the multiplex ctDNA assay we investigated the association between treatment response and the presence of ctDNA during and after neoadjuvant breast cancer treatment (independent of randomization group). There was no association between ctDNA status and pCR (RCB 0), neither midterm (*p* = 0.06) nor preoperatively (*p* = 0.6). No baseline samples were available for ctDNA analysis, which is a limitation of this study.

A recent meta-analysis including six studies did not demonstrate any association between the presence of ctDNA at baseline and the achievement of pCR, but in three of the studies the presence of ctDNA 2–3 weeks after initiation of neoadjuvant breast cancer treatment was associated with lower pCR rates^9^. Another study (not part of the above meta-analysis) reported the presence of ctDNA at midterm to be associated with poor response to neoadjuvant treatment defined as RCB II/III^8^. In order to compare these findings with ours, we conducted a post hoc analysis using response classification according to Zhou et al. (RCB 0–I vs. II–III). This resulted in an association between response to neoadjuvant treatment and ctDNA status at midterm (*p* = 0.012), which is in line with the findings by Zhou et al. No association between treatment response and preoperative ctDNA status was observed (*p* = 0.79).

It has previously been reported that presence of ctDNA during and after completion of neoadjuvant breast cancer treatment associates with poor survival^9^. The final aim of this study was to evaluate if ctDNA status holds prognostic information. According to the results, ctDNA status was not associated with OS and IDFS, neither at midterm nor before or after surgery. Thus, in contrast to previous studies^8,9^, we did not identify a prognostic value of ctDNA evaluation during and after neoadjuvant breast cancer treatment. However, due to the very few events during the study period, the survival analysis should be interpreted with caution. Also, since the study was designed with the main aim of evaluating the effect of tocotrienol, the size of the study population might be inadequate for the ctDNA analyses, and although the type two error rate was 20% for the main hypothesis, a lower statistical power cannot be ruled out for the ctDNA analyses.

In conclusion, delta-tocotrienol did not enhance the efficacy of neoadjuvant breast cancer treatment and did not reduce the frequency of side effects. The results showed no association between the presence of ctDNA and response to neoadjuvant treatment. The sensitivity of the ctDNA assay can be increased by targeting a combination of tissue specific and cancer specific markers and by applying sense and antisense analyses, which allowed for detection of ctDNA in half of the patients. According to existing literature, ctDNA holds the potential of serving as early markers of response to neoadjuvant breast cancer treatment, but there is an obvious need for well planned prospective, preferably randomized trials with high analytical sensitivity.

## Supplementary Information

Supplementary Information.

## Data Availability

The dataset contains person sensitive data used under license for the study and are only available with permission from the relevant legal authorities and according to existing regulations. For further information please contact the corresponding author.
