# Supplementary material for: Phase II trial of delta-tocotrienol in neoadjuvant breast cancer with evaluation of treatment response using ctDNA
Source: Sci Rep. 2023 May 24;13:8419. doi: 10.1038/s41598-023-35362-7 (PMC10209064; doi:10.1038/s41598-023-35362-7)
Supplement: Supplementary file 1 — Supplementary Information. [file 41598_2023_35362_MOESM1_ESM.docx]

**Supplementary material: Phase II trial of delta-tocotrienol in neoadjuvant breast cancer with evaluation of treatment response using ctDNA**

**Authors**

Ina Mathilde Kjær^1,3^, Søren Kahns^1^, Signe Timm^2,3^, Rikke Fredslund Andersen^1,2^, Jonna Skov Madsen^1,3^, Erik Hugger Jakobsen^2,4^, Tomasz Piotr Tabor^5,6^, Anders Jakobsen^2,3^, Troels Bechmann^2,7^

**Affiliations**

^1^Department of Biochemistry and Immunology, Vejle Hospital, University Hospital of Southern Denmark, Denmark

^2^Department of Oncology, Vejle Hospital, University Hospital of Southern Denmark, Denmark

^3^Department of Regional Health Research, Faculty of Health Sciences, University of Southern Denmark, Denmark

^4^Department of Medicine, Hospital Soenderjylland, University Hospital of Southern Denmark, Denmark

^5^Department of Pathology, Vejle Hospital, University Hospital of Southern Denmark, Denmark

^6^Department of Pathology, Viborg Hospital, Regional Hospital Central Jutland, Denmark

^7^Department of Oncology, Regional Hospital West Jutland, Denmark

*Corresponding author: ina.mathilde.kjaer@rsyd.dk

**Kaplan-Meier survival functions depicting overall survival (OS) and invasive disease free survival (IDFS) for patients included in the NeoToc study in relation to CtDNA status at midterm, preoperatively and postoperatively.**


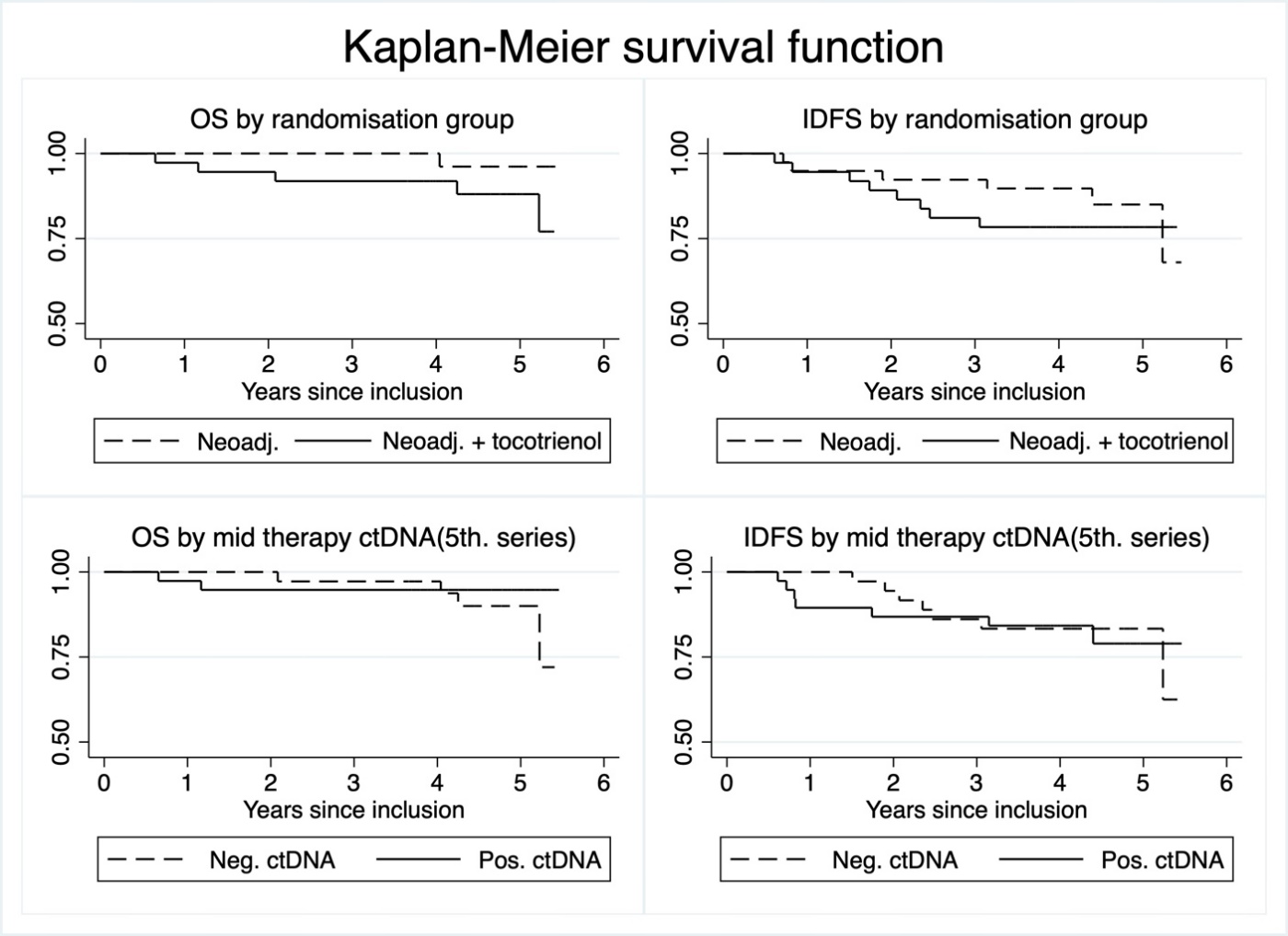

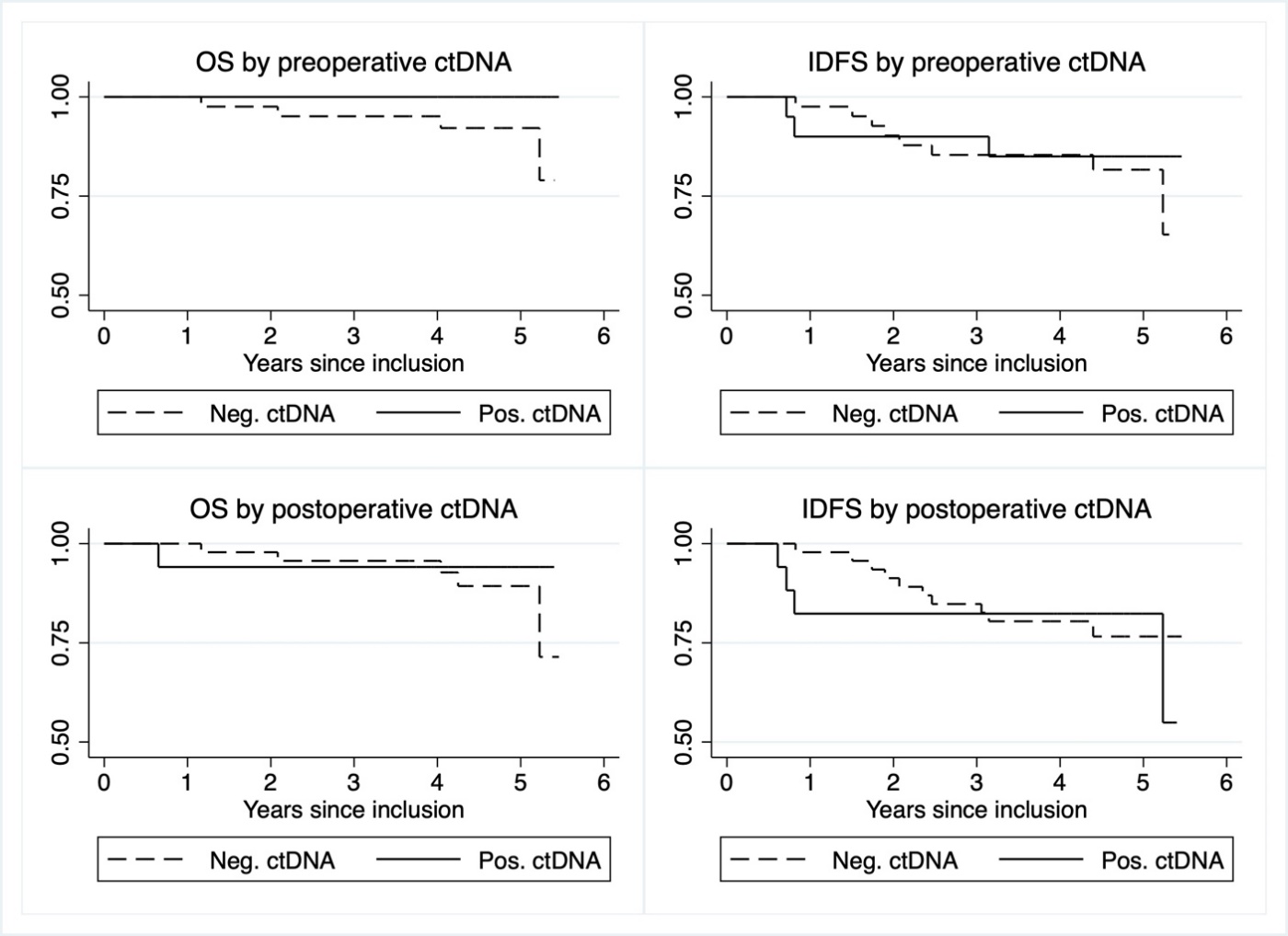


P_log rank_ = 0.96

P_log rank_ = 0.38

P_log rank_ = 0.64

P_log rank_ = 0.14

P_log rank_ = 0.88

P_log rank_ = 0.46

**Design of breast epithelium methylation specific primers and probes**

Three genomic regions (*LMX1B*, *KRT19*, and *ZNF296*) have been reported to display unique methylation patterns in breast epithelium [1]. We designed primers and probes (Table S1) to target bisulfite converted DNA with the breast specific methylation patterns observed in the *LMX1B*, *KRT19*, and *ZNF296* regions, respectively. Primers and probes were designed in order to keep amplified fragments as short as possible (Table S1). We were not able to design an assay specific for hypomethylated converted DNA targeting the *KRT19* region (data not shown). Since we have previously shown that targeting bisulfite converted sense and antisense DNA increases the sensitivity of the *HOXA9* ddPCR assay [2], the primers and probes were also designed to target the converted antisense DNA region of *LMX1B*. We did not succeed in designing a specific antisense *ZNF296* assay.

**Design of a breast cancer multiplex droplet digital PCR assay**

We designed a multiplex ddPCR assay targeting bisulfite converted DNA specific for 1) breast epithelium cells (*LMX1B* sense + *LMX1B* antisense + *ZNF296* sense assays) and 2) a general marker of cancer (*HOXA9* sense + *HOXA9* antisense). Furthermore, the assay targeted the endogenous control (*ALB*). The *LMX1B* sense + *LMX1B* antisense assay formed one delimited ddPCR cloud in the 2D amplitude plot (Figure S1). The *HOXA9* sense + *HOXA9* antisense assay also formed one delimited ddPCR cloud whereas the *ZNF296* assay only included a sense part forming one cloud.

Five point three-fold dilution series of extracted and bisulfite converted DNA from breast tumor biopsies was used as template in ddPCR analyses to compare performance of the *LMX1B* sense assay alone with the performance of sense + antisense assays in combination (Figure S2). A similar experimental setup was used to verify that an assay displayed similar performance being part of the multiplex assay as well as in combination with the reference *ALB* gene alone (Figure S2).

Samples were analyzed on the QX200 Droplet Digital PCR System (Bio-Rad) according to the manufacturer’s instructions and analyzed using the QX Manager Standard Edition, version 1.1 (BioRad) software.

**Specificity and limit of blank (LOB)**

The specificity of the multiplex assay was tested on bisulfite converted DNA extracted from normal whole blood and breast tissues (Figure S3). The assay was tested on bisulfite converted DNA extracted from breast cancer, colon cancer, lung cancer and ovarian cancer tissues.

The LOB for *HOXA9* (sense+antisense) was previously determined to four positive droplets [2]. The LOB of *LMX1B* (sense+antisense) and *ZNF296* was determined from analysis of plasma from healthy female donors. Plasma samples were divided into two groups. Twenty plasma samples from healthy donors were initially analyzed by the multiplex assay and the number of positive droplets counted. Based on the results the LOB was set to one positive droplet for *LMX1B* and one positive droplet for *ZNF296*. The results were subsequently validated by analyzing another 20 plasma samples from healthy donors. Except for two positive droplets detected in one sample of the *LMX1B* target, the results were similar. With a 95% confidence limit this sets the LOB to one positive *LMX1B* droplet and one positive *ZNF296* droplet. The cut-off values for positivity were set at two positive *LMX1B* droplets, two positive *ZNF296* droplet, and five positive *HOXA9* droplets.

The ctDNA positivity of a blood sample was defined as the presence of a positive signal for one or more of the targets described above.

**Table S1. Primers and probes used**

**Figure S1. 2D amplitude plots**

Examples of 2-dimensional QX Manager amplification plots of the multiplex assay for the analysis of bisulfite converted DNA of A) hypermethylated human genomic control DNA, B) hypomethylated human genomic control DNA, C) DNA extracted from breast tissue, and D) cell free DNA extracted from plasma from patients with breast cancer. Clusters, defined according to plots from bisulfite converted control DNA, are indicated in identical coloring as droplet and name of target gene. Channel 1 is FAM. Channel 2 is VIC.
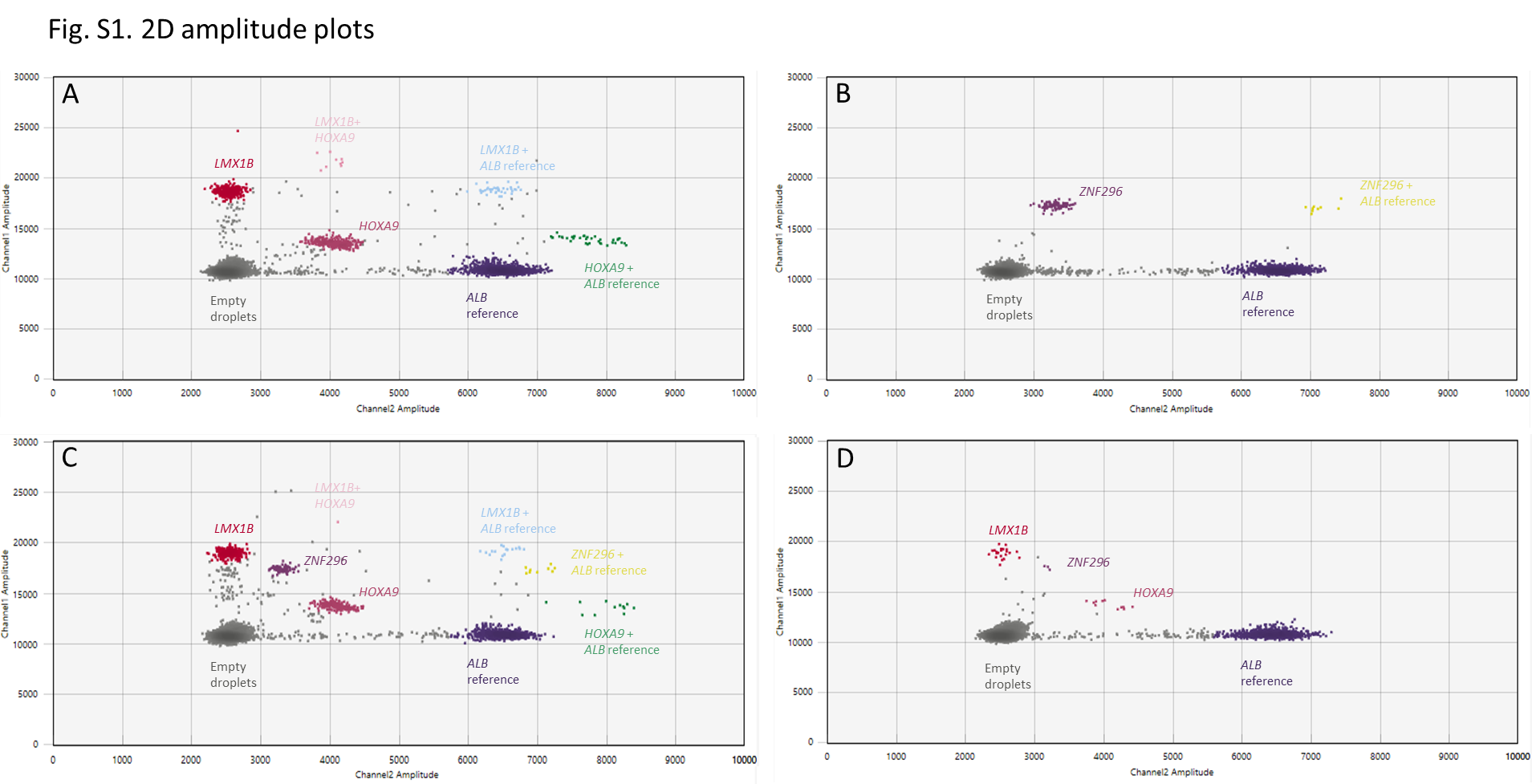


**Figure S2. Correlation between assays in multiplex assay**

Comparison of the performance of the LMX1B, ZNF296 and HOXA9 DNA methylation markers in single plex/duplex and multiplex reactions. All assays included the albumin reference as well. Five point three fold dilution series of extracted bisulfite converted DNA from a breast tumor biopsy was used as template. An NTC was included in each cycle. s: sense; as: antisense. The numbers on the axes correspond to the number of target molecules in 20 μl reaction.


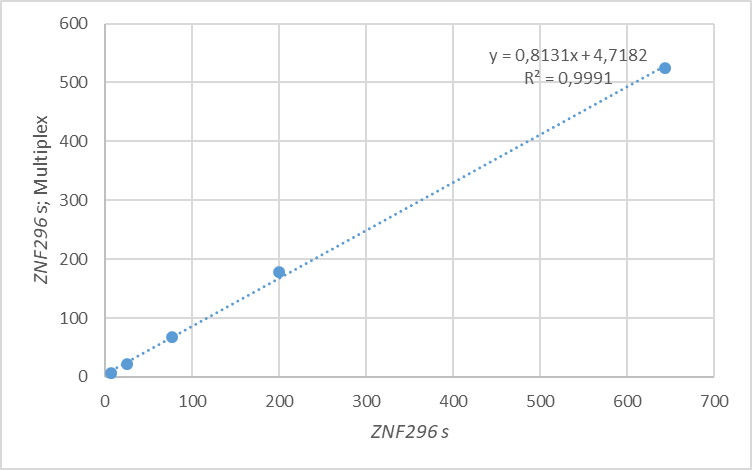

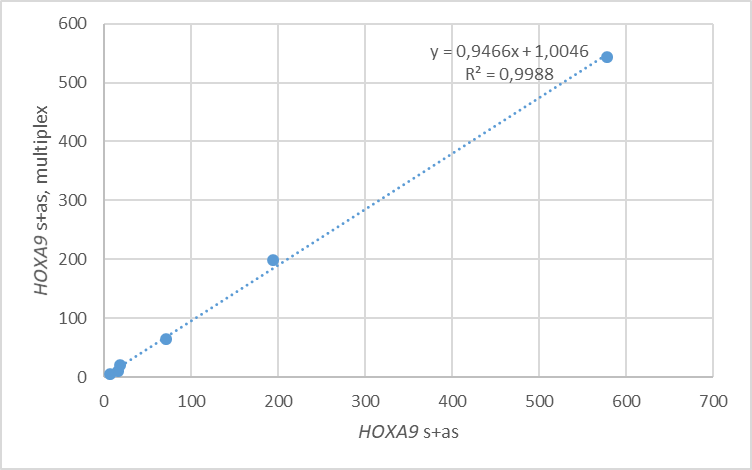

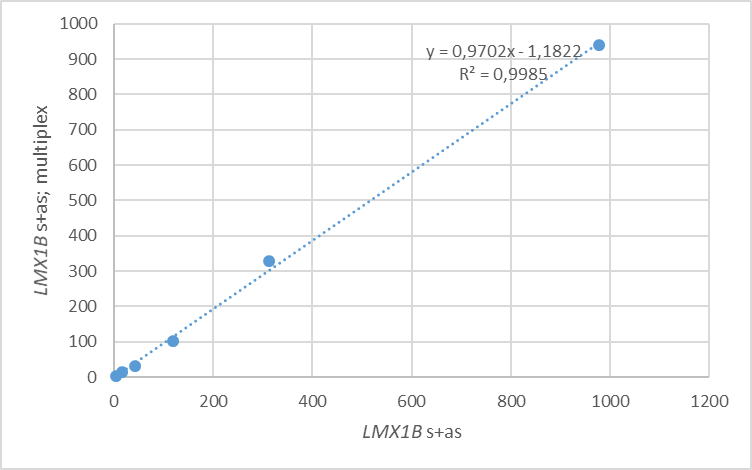

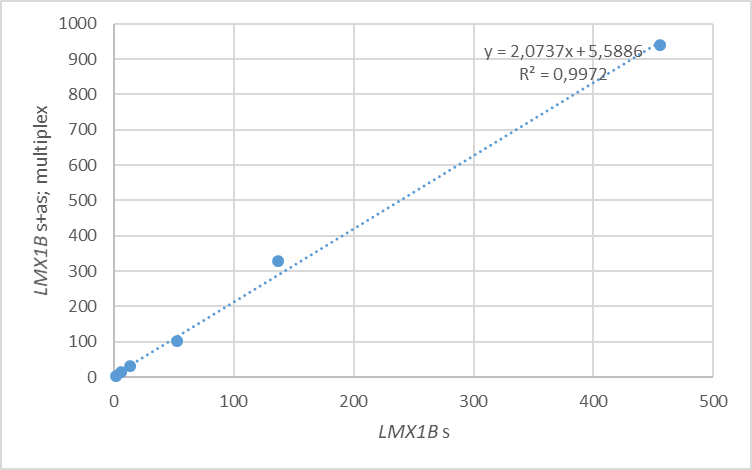


**Figure S3. Specificity in normal and cancer tissues**

A: The multiplex assay was tested on bisulfite converted DNA extracted from normal blood (B) and breast (M) tissue. B: The multiplex assay was tested on bisulfite converted DNA extracted from breast cancer (MT), colon cancer (C), lung cancer (L) and ovarian cancer (O) tissues. Results are illustrated as ratios relative to the albumin reference gene. ZNF296 ratio=blue, LMX1B ratio=orange, HOXA9 ratio=grey.


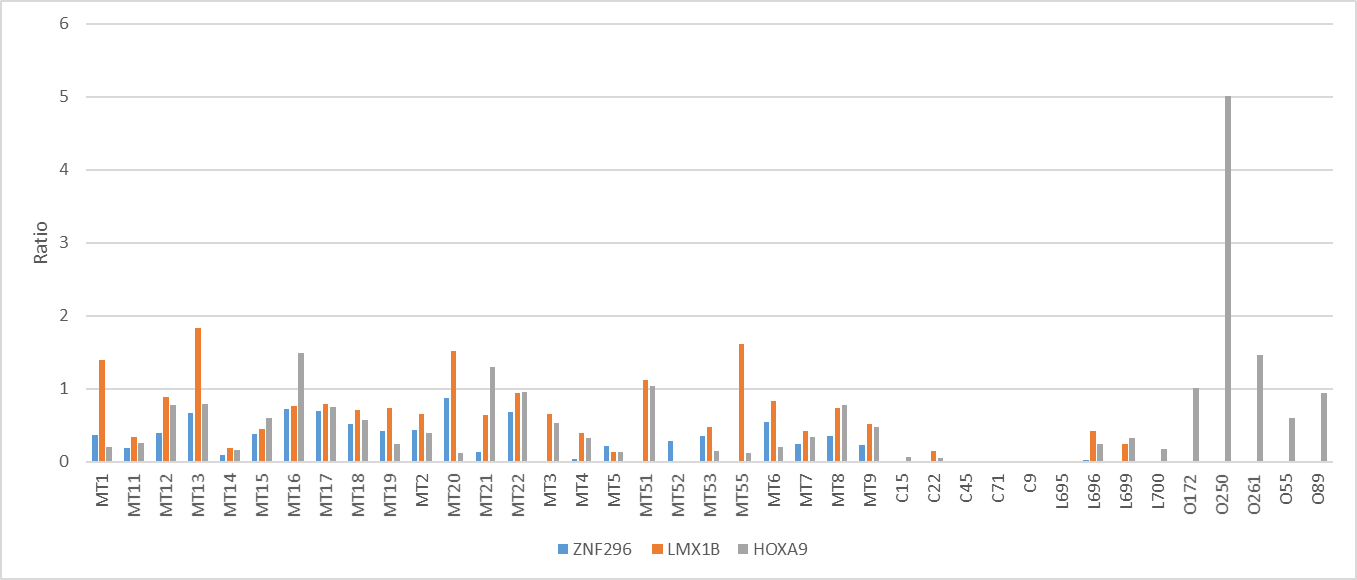

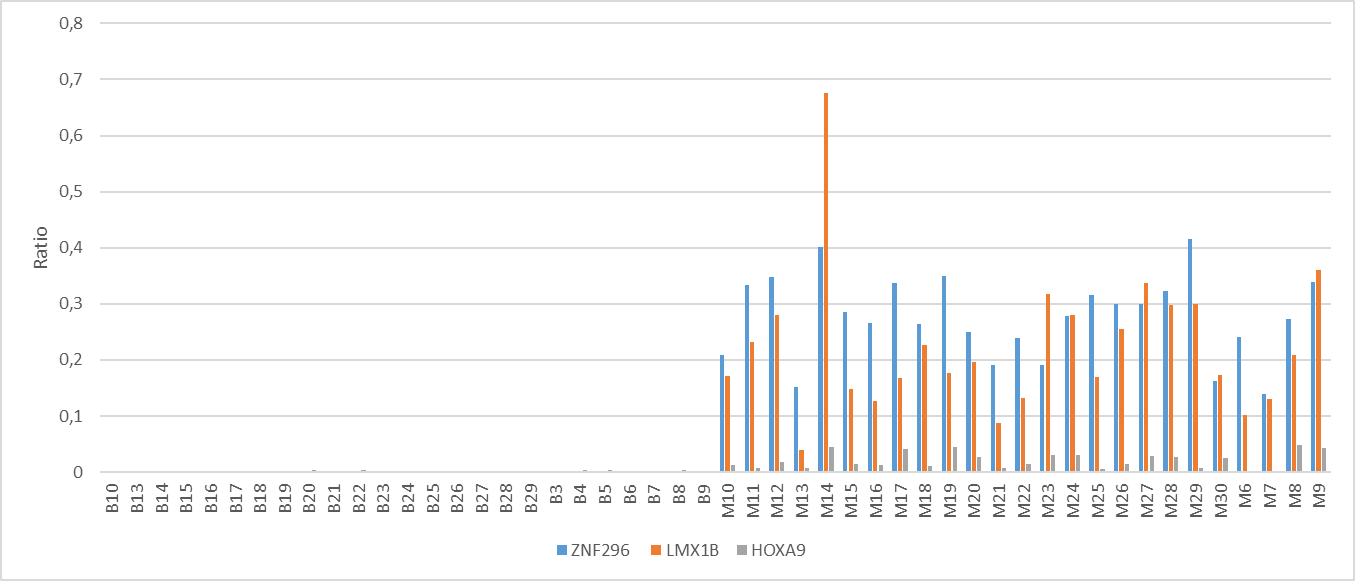


A

B

**Figure S4. LOB in plasma samples from healthy women**

The multiplex assay was tested on plasma from healthy women. Initially, 20 plasma samples were analyzed (A-C). Results were then validated on plasma samples from another 20 women (D-E). A and D show LMX1B positive droplets whereas B and E, and C and F show the number of ZNF296 and HOXA9 positive droplets, respectively.

A

B

C


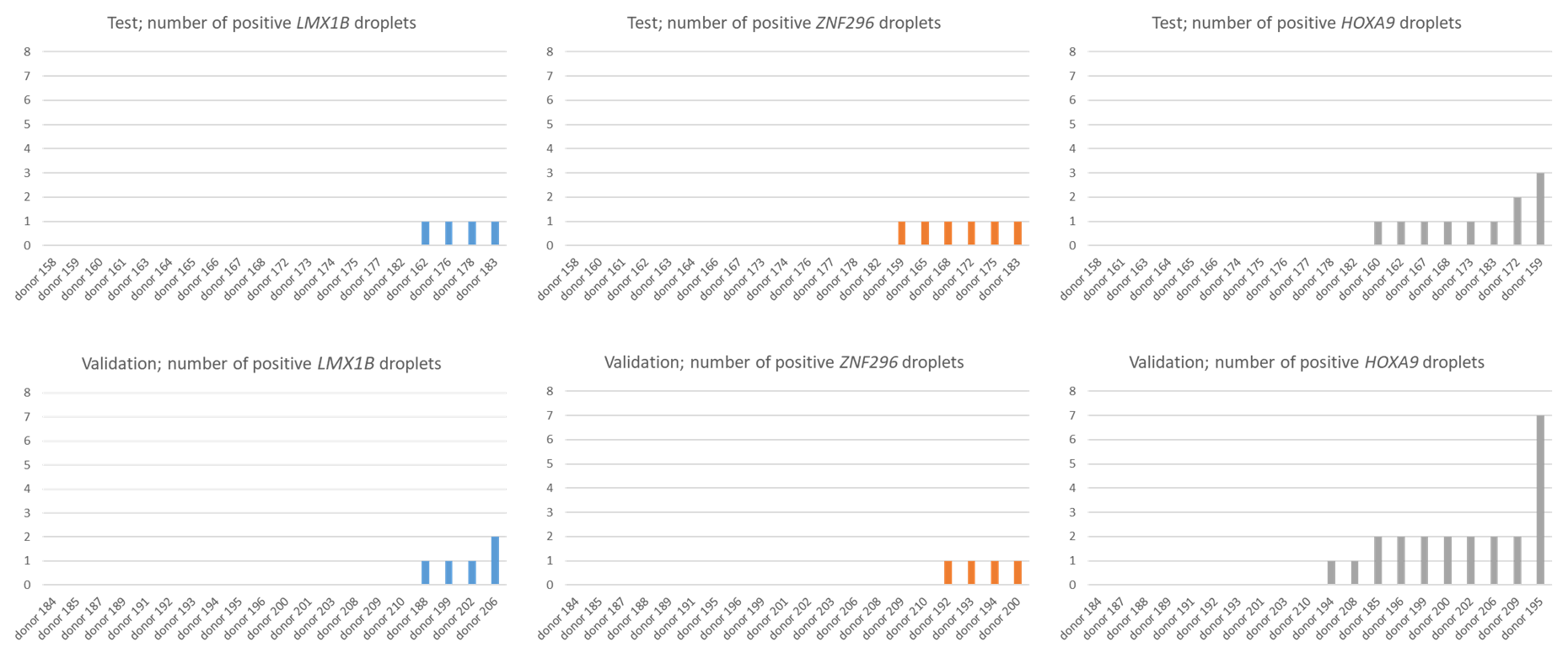


C

D

E

**References**

1. Moss J, Zick A, Grinshpun A, Carmon E, Maoz M, Ochana BL, et al. Circulating breast-derived DNA allows universal detection and monitoring of localized breast cancer. Annals of oncology : official journal of the European Society for Medical Oncology / ESMO. 2020;31(3):395-403.

2. Faaborg L, Fredslund Andersen R, Waldstrom M, Hogdall E, Hogdall C, Adimi P, et al. Analysis of HOXA9 methylated ctDNA in ovarian cancer using sense-antisense measurement. Clin Chim Acta. 2021;522:152-7.
